# Association between promoter -1607 polymorphism of *MMP1 *and Lumbar Disc Disease in Southern Chinese

**DOI:** 10.1186/1471-2350-9-38

**Published:** 2008-04-28

**Authors:** You-Qiang Song, Daniel WH Ho, Jaro Karppinen, Patrick YP Kao, Bao-Jian Fan, Keith DK Luk, Shea-Ping Yip, John CY Leong, Kathryn SE Cheah, Pak Sham, Danny Chan, Kenneth MC Cheung

**Affiliations:** 1 Department of Biochemistry, University of Hong Kong, Hong Kong, China; 2 Department of Orthopaedics and Traumatology, University of Hong Kong, Hong Kong, China; 3 Department of Rehabilitation, University of Oulu, Oulu, Finland; 4 Department of Health Technology and Informatics, The Hong Kong Polytechnic University, Hong Kong, China; 5 Department of Psychiatry, University of Hong Kong, Hong Kong, China; 6 Open University of Hong Kong, Hong Kong, China

## Abstract

**Background:**

Matrix metalloproteinases (MMPs) are involved in the degradation of the extracellular matrix of the intervertebral disc. A SNP for guanine insertion/deletion (G/D), the -1607 promoter polymorphism, of the *MMP1 *gene was found significantly affecting promoter activity and corresponding transcription level. Hence it is a good candidate for genetic studies in DDD.

**Methods:**

Southern Chinese volunteers between 18 and 55 years were recruited from the population. DDD in the lumbar spine was defined by MRI using Schneiderman's classification. Genomic DNA was isolated from the leukocytes and genotyping was performed using the Sequenom^® ^platform. Association and Hardy-Weinberg equilibrium checking were assessed by Chi-square test and Mann-Whitney U test.

**Results:**

Our results showed substantial evidence of association between -1607 promoter polymorphism of *MMP1 *and DDD in the Southern Chinese subjects. D allelic was significantly associated with DDD (p value = 0.027, odds ratio = 1.41 with 95% CI = 1.04–1.90) while Genotypic association on the presence of D allele was also significantly associated with DDD (p value = 0.046, odds ratio = 1.50 with 95% CI = 1.01–2.24). Further age stratification showed significant genotypic as well as allelic association in the group of over 40 years (genotypic: p value = 0.035, odds ratio = 1.617 with 95% CI = 1.033–2.529; allelic: p value = 0.033, odds ratio = 1.445 with 95% CI = 1.029–2.029). Disc bulge, annular tears and the Schmorl's nodes were not associated with the D allele.

**Conclusion:**

We demonstrated that individuals with the presence of D allele for the -1607 promoter polymorphism of *MMP1 *are about 1.5 times more susceptible to develop DDD when compared with those having G allele only. Further association was identified in individuals over 40 years of age. Disc bulge, annular tear as well as Schmorl's nodes were not associated with this polymorphism.

## Background

Low back pain (LBP) affects 70% to 80% of all people at some time during their life [1]. The annual prevalence ranges from 15% to 45%, with a point prevalence averaging 30% [1]. Degenerative Disc Disease (DDD) in the lumbar spine or lumbar disc degeneration is a major cause of LBP [2-7].

Up to now, there is only a limited understanding of DDD, with the underlying pathophysiological and molecular mechanisms remain un-elucidated. Studies suggested that environmental factors such as physical loading, motor vehicle driving, vibration and smoking may have a role [8-11]. However, there is increasing evidence that DDD is a genetic disorder [12], with reports of predisposition in association with certain genes, such as *Taq *I polymorphism of vitamin D receptor (*VDR*) gene [2,5,13,14], polymorphism of metalloproteinase-3 (*MMP3*) gene [15-17], mutations in collagen IX genes (*COL9A2 *and *COL9A3*) [18-22], Sp1 polymorphism of collagen I gene (*COL1A1*) [23,24], polymorphism of cartilage intermediate layer protein (*CILP*) [25] as well as aggrecan gene polymorphism [26]. The involvement of multiple genes is not uncommon, as aetiology is often multifactorial in common diseases such as DDD [12].

In the process of disc degeneration, degradation of the extracellular matrix (ECM) is a key event. Collagens are one of the major constituents of the ECM. They make up 20% dry mass of the nucleus pulposus and 60% dry mass of the annulus fibrosis. Approximately 80% of the collagens in the intervertebral disc consist of fibrillar collagen types I and II [16,27]. Matrix metalloproteinases (MMPs) are the family of degradation enzymes that break down the components of the ECM [17,27,28]. In particular, MMP1 is a collagenase which cleaves the triple helical part of the fibrillar collagen of types I, II and III, and is proposed to initiate the degradation process [29-35]. The cleaved fragments have a lower melting temperature than 37°C, which are then further cleaved by other MMPs. Similarly, MMP1 can cleave aggrecan at the major MMP cleavage sites between the G1 and G2 domains contributing to the breakdown of aggrecan [36].

Expression of MMPs is generally low in normal cells which allows for healthy tissue turnover. On the other hand, the level of MMPs increases substantially under pathological conditions and results in ECM degradation. A single nucleotide polymorphism (SNP) for guanine insertion/deletion (G/D) at position -1607 in the promoter region of the *MMP1 *gene (-1607 polymorphism) results in creation of binding sites for Ets transcription factor family [29]. Upon the interaction of adjacent AP-1 site, the promoter activity and hence the transcription level of *MMP1 *can be considerably increased [29,30].

In the present study, the association between this promoter SNP and DDD was investigated in a population-based dataset [14,22]. We demonstrated significant association with DDD and the deletion of guanine is suggested to be a possible genetic risk factor predisposing to DDD.

## Methods

### Subjects & assessment

The study was approved by the local ethics committee and informed consent was obtained. Individuals of Southern Chinese origin were recruited by open invitation from the general population. All the recruited subjects subsequently underwent magnetic resonance imaging (MRI) of the lumbar spine and their blood was obtained for DNA extraction and genotyping analysis.

The MRI examinations were performed at the Jockey Club MRI Engineering Centre using a 0.2T Profile open MRI system (General Electric Medical System, Milwaukee, WI). Sagittal T2-weighted fast spin echo sequences (TR = 3000 ms, TE = 92 ms, slice thickness = 5 mm) were used to image the lumbar spine with a built-in flexible body coil. Despite the relative low field, previous studies have demonstrated this to be sensitive enough to identify abnormalities associated with DDD [14,22].

DDD was diagnosed on the basis of signal intensity changes within the nucleus pulposus (NP) of the intervertebral discs (IVDs) of the lumbar spine and graded using the Schneiderman's classification scheme [37]. Grade 0 was used to indicate normal disc with hyperintense signal within the NP, grade 1 for a slight decrease in signal intensity in the NP, grade 2 for a generalized hypointense NP and grade 3 for a hypointense NP with disc space narrowing. All the MRI scans were analyzed and rated by two experienced physicians blinded to the results of the genetic analysis and clinical history. Differences were reviewed by the two raters and settled by consensus. The score for each disc (0–3) was then summated to create a DDD score for the whole lumbar spine (0–15) as previously described [14]. To define the affection status using DDD score, a score of 0 was used to indicate the absence of disc degeneration, while a score of 2 or above was used to indicate the presence of degeneration. Individuals with DDD score of 1 are considered as borderline degeneration and was the area of the majority of disagreements between the 2 raters and hence those with DDD score of 1 was considered unknown affection status and excluded from analysis [14,22].

Disc bulge was defined in discs where it has protruded posteriorly beyond a line connecting the posterior margins of the adjacent vertebral bodies. In addition, annular tears and Schmorl's nodes were assessed [14]. Annular tears were defined as areas of high signal intensity within the posterior annulus surrounded by a dark rim. Schmorl's nodes were defined as areas of endplate irregularities in which the darkened rim of the vertebral endplate has indented into the vertebral body.

### SNP genotyping

Genomic DNA was isolated from the leukocytes and the quality as well as quantity measured by spectrophotometry. Genotyping was performed using the Sequenom^® ^platform. The Mass ARRAY AssayDesign software (Sequenom) was used to design amplification and allele-specific extension primers. The extension primer (5'-**GTAGTTAAATAATTAGAAAG-3') **was designed to hybridize to the amplicon near the SNP site for the extension of a single base or a few bases depending on the genotype of the allele. PCR reactions (Forward primer: 5'-**ACGTTGGATGGAACTCACATGTTATGCCAC-3'; **Reverse-primer: 5'-**ACGTTGGATGCTTCAGTATATCTTGGATTG-3'**) were set up in 384-well plates at 6 μL total volume per reaction and the reaction mix contains: 5 ng genomic DNA, 0.3 pmol each of specific forward and reverse primers, 200 μM of each dNTP, 3.25 mM MgCl_2 _and 0.2 unit of HotStarTaq polymerase (5U/μL, Qiagen, Valencia, CA). The PCR condition was: 95°C for 15 min, 45 cycles of 95°C for 20 sec, 56°C for 30 sec and 72°C for 1 min, followed by 72°C for 3 min. The treatment of PCR products with alkaline phosphatase and mass extend reactions were all performed according to manufacturer's (Sequenom) protocol. The final base-extension products were desalted using SpectroClean resin (Sequenom), mixed with 3-hydroxypicolinic acid and analyzed using a modified Brucker Autoflex MALDI-TOF mass spectrometer (Brucker, Billerica, MA).

### Statistical analysis

Association and Hardy-Weinberg equilibrium checking were assessed by χ^2 ^test and Mann-Whitney U test. Odds ratios and other statistical measures were calculated by SPSS 14.0 software.

## Results

A total of 691 individuals were genotyped for the study with 266 males and 425 females. In common with previous studies, no significant differences could be detected in disease predisposition between the 2 genders and therefore no gender stratification was carried out [11,13,14].

Analysis of the D allele showed an over representation in the case group. The D allele conferred a higher risk (1.4 times) of developing DDD when compared to the G allele (*P*-value = 0.027, odds ratio = 1.41 with 95% CI = 1.04–1.90) (Table 1). Genotypic association on the presence of the D allele was also significantly associated with DDD (*P*-value = 0.046, odds ratio = 1.50 with 95% CI = 1.01–2.24) (Table 2).

**Table 1:** Allelic association of DDD with the -1607 polymorphism of *MMP1*

	Total		Age ≤ 40		Age > 40	
	G (%)	D (%)	G (%)	D (%)	G (%)	D (%)

Control	170 (69.7)	74 (30.3)	31 (67.4)	15 (32.6)	139 (70.2)	59 (29.8)
Case	593 (62.0)	363 (38.0)	143 (62.2)	87 (37.8)	450 (62.0)	276 (38.0)
OR (95% CI)	1.41 (1.04–1.90)		1.26 (0.64–2.46)		1.46 (1.03–2.03)	
p value	0.027		0.503		0.033	

Note: The G allele is the reference allele of the calculation of ORs.

**Table 2:** Genotypic association of LDD with the -1607 polymorphism of *MMP1*

	Total		Age ≤ 40		Age > 40	
	GG (%)	DD/DG (%)	GG (%)	DD/DG (%)	GG (%)	DD/DG (%)

Control	58 (47.5)	64 (52.5)	9 (39.1)	14 (60.9)	49 (49.5)	50 (50.5)
Case	180 (37.7)	298 (62.3)	43 (37.4)	72 (62.6)	137 (37.7)	226 (62.3)
OR (95% CI)	1.50 (1.01–2.24)		1.08 (0.43–2.70)		1.62 (1.03–2.53)	
p value	0.046		0.875		0.035	

Note: The GG genotype is reference genotype of the calculation of ORs.

Because DDD is age related and 2 risk factors, *VDR *[14] and the *COL9A2 *[22], have previously been demonstrated to be age-dependent, we also analyzed this *MMP1 *association to DDD stratified by age group. Using a stratification as in a previous study [14], no significant association was detected in an under-40-year age group (*P*-value = 0.5, OR = 1.26 with 95% CI = 0.64–2.46). However, in the over-40-year age group, the association was significant (*P*-value = 0.03, OR = 1.46 with 95% CI = 1.03–2.03) (Tables 1 &2).

In addition, we assessed the difference in the distribution of the DDD score for the D-allele+ (either DD or DG genotype) and D-allele- (GG genotype) groups by Mann-Whitney U test. It was found that the distribution of the DDD score was significantly different between the D-allele+ and D-allele- groups (p value = 0.028) with D-allele+ group having higher DDD score.

Apart from DDD, association was tested on disc bulge, annular tear as well as Schmorl's nodes, no significant association with these phenotypes were found with the *MMP1 *promoter polymorphism (Table 3).

**Table 3:** Genotypic association of other degenerative changes with the -1607 polymorphism of *MMP1*

		GG (%)	DD/DG (%)	p value
Disc bulge	Control	122 (42.7)	164 (57.3)	0.316
	Case	113 (38.6)	180 (61.4)	
Annular tear	Control	215 (41.0)	309 (59.0)	0.430
	Case	55 (37.4)	92 (62.6)	
Schmorl's node	Control	239 (40.0)	358 (60.0)	0.758
	Case	31 (41.9)	43 (58.1)	

## Discussion

This is the first report of an association of a SNP in the promoter region of *MMP1 *with DDD. DDD is a complex multifactorial disease. It has been suggested that disc degeneration is highly correlated to age [15,38-41] and age stratification was performed in previous studies [14,22]. Our results indicated that the association of DDD with the SNP is age-dependent, and is associated with subjects over 40 years of age.

MMP1 is involved in the cleavage of fibrillar collagens I, II and III [29-35]. Type I collagen is found extensively within the annulus fibrosus and bony end-plate, while Type II is the predominant collagen in the inner annulus, nucleus pulposus as well as the cartilaginous end-plate [16,27]. Previous studies in tumor cell lines showed that an over-representation of the G allele of this promoter SNP resulted in increased *MMP1 *transcriptional activity, hence more aggressive matrix degradation [29]. This was proposed to be a mechanism in cancer progression and was proven in subsequent functional studies which showed that the G allele created additional binding sites for the Ets family of transcription factors [29]. These Ets binding sites (-1607 bp) work cooperatively with a nearby AP-1 site (-1602 bp) to enhance promoter activity by at least 2 folds [29,30]. With the increased promoter activity and hence transcription level, the presence of the G allele could consider to be risk factor for matrix degradation.

Although we found an association with this promoter SNP in *MMP1*, our result showed the presence of the D allele resulted in an increased risk of DDD, and not the anticipated G allele. Previous *in vitro *studies have shown that the G allele is associated with higher expression levels of *MMP1 *[29,30]. Thus, individuals with the D allele with increase risk for DDD could not be explained by increased susceptibility to matrix degradation because there is no over-expression of *MMP1*.

This association may be explained in a number of ways. First, the frequency for D allele is high in different ethnic populations with 62.5% and 56.7% for African and Caucasian respectively (NCBI database), and 36.4% for Southern Chinese. The high frequency of D allele may indicate that the D allele is a common ancestor allele, which might be in linkage disequilibrium with unknown disease-causing polymorphism within the same gene. Secondly, *MMP1 *is located in a gene cluster on chromosome 11 with other *MMP *genes (*MMP-3, 7, 8, 10, 12, 13, 20 *and *26*). It is possible that the observed association is the result of linkage disequilibrium between this *MMP1 *promoter polymorphism and polymorphisms in other nearby MMP genes. Moreover, despite the Ets binding site can cooperate with AP-1 site to enhance transcription of *MMP1*, this polymorphism has yet to be tested in intervertebral disc tissues, to see whether it affects the transcription level of *MMP1*. It maybe that gene expression be differentially displayed in a tissue-specific manner. In addition, expressional activity of *MMP1 *may also be influenced differentially in response to cytokines and growth factors. Even in the case of cancer, allele corresponding to elevated *MMP1 *activity (expected to have increased susceptibility to cancer and poor prognosis) was unexpectedly found to have favourable prognosis with colorectal cancer and no association with tumor characteristics [42]. Thus further replications of association in other ethnic populations would help substantiate findings in this study.

## Conclusion

In summary, we have performed a case-control association study to test for association between this promoter SNP (-1607 polymorphism) of MMP1 and DDD. We have demonstrated this promoter polymorphism was significantly associated with increased susceptibility to DDD in our Southern Chinese population, especially in individuals over 40 years of age. Disc bulge, annular tear as well as Schmorl's nodes were not associated with this polymorphism.

## Competing interests

The authors declare that they have no competing interests.

## Authors' contributions

YQ, DW, PYP and BJ carried out molecular studies. YQ, DW and KMC drafted the manuscript. JK, KMC, KDK and JCY carried out clinic studies. DW, SPY and PS performed the statistical analysis. YQ, KSE, DC and KMC conceived of the study, and participated in its design and coordination. All authors read and approved the final manuscript.

## Pre-publication history

The pre-publication history for this paper can be accessed here:


